# Ophiopogonin D Increases SERCA2a Interaction with Phospholamban by Promoting CYP2J3 Upregulation

**DOI:** 10.1155/2020/8857906

**Published:** 2020-12-31

**Authors:** Jia Wang, Wenting You, Ningning Wang, Wei Zhou, Yunxuan Ge, Zengchun Ma, Hongling Tan, Yuguang Wang, Yue Gao

**Affiliations:** ^1^Department of Pharmaceutical Sciences, Beijing Institute of Radiation Medicine, Beijing 100850, China; ^2^Guangdong Pharmaceutical University, Guangzhou 510006, China; ^3^Department of Pharmacology, Anhui Medical University, Hefei 230032, China

## Abstract

Ophiopogonin D (OPD), a compound from the Chinese herb Radix Ophiopogonis, reportedly induces increased levels of cytochrome P450 2J3 (CYP2J3)/epoxyeicosatrienoic acids (EETs) and Ca^2+^ in rat cardiomyocytes. Little is known regarding the specific mechanism between CYP2J3 and Ca^2+^ homeostasis. Here, we investigated whether CYP2J3 is involved in the protective action of OPD on the myocardium by activating the Ca^2+^ homeostasis-related protein complex (SERCA2a and PLB) in H9c2 rat cardiomyoblast cells. The interaction between SERCA2a and PLB was measured using fluorescence resonance energy transfer. OPD attenuated heart failure and catalyzed the active transport of Ca^2+^ into the sarcoplasmic reticulum by inducing the phosphorylation of PLB and promoting the SERCA2a activity. These beneficial effects of OPD on heart failure were abolished after knockdown of CYP2J3 in a model of heart failure. Together, our results identify CYP2J3 as a critical intracellular target for OPD and unravel a mechanism of CYP2J3-dependent regulation of intracellular Ca^2+^.

## 1. Introduction

Cytochrome P450 2J3 (CYP2J3) is a member of the cytochrome P450 superfamily of enzymes and catalyzes many reactions involved in the metabolism of drugs and other xenobiotics. They are highly expressed in the myocardium and are involved in the metabolism of arachidonic acid (AA). The metabolites of AA are epoxyeicosatrienoic acids (EETs), which reportedly have anti arrhythmic [1], anti-inflammatory [2], antiapoptotic [3], and antioxidant [4] effects, as well as a role in cardiovascular protection [5]. Specifically, EETs can maintain Ca^2+^ homeostasis in myocardial cells and alleviate the symptoms of heart failure in rats by upregulating the expression of sarcoplasmic reticulum Ca^2+^-ATPase (SERCA) and phospholamban (PLB). Additionally, the CYP2J3 overexpression and increased levels of EETs reportedly decrease endoplasmic reticulum (ER) stress signaling and ER stress-mediated apoptosis in rats with heart failure (HF) by maintaining the sarcoplasmic reticulum Ca^2+^-ATPase (SERCA2a) activity and intracellular Ca^2+^ homeostasis [6].

Research has indicated that the decreased SERCA2a activity and expression are hallmarks of HF in both experimental animal models and patients [7, 8]. SERCA2a regulates Ca^2+^ reuptake into the sarcoplasmic reticulum (SR), and dysregulated Ca^2+^ homeostasis is an initiating event in HF [9]. Furthermore, the SERCA2a activity can be regulated by a small intrinsic protein located in the SR named phospholamban (PLB). In the dephosphorylated state, PLB inhibits the SERCA2a activity and SR Ca^2+^transport. Phosphorylation of PLB by either cAMP-dependent protein kinase (PKA) at Ser^16^ residue or Ca^2+^-calmodulin-dependent protein kinase (CaMKII) at Thr^17^ residue relieves the inhibition of the SERCA2a activity, thus increasing the rate of SR Ca^2+^ uptake [10]. As a result, the SERCA2a-PLB Ca^2+^-regulatory system has been implicated in cardiovascular disease [11, 12]. Industry and academic researchers have extensively searched for the affinity of the regulatory interaction between SERCA2a and PLB. For instance, fluorescence resonance energy transfer (FRET) has been used to directly detect the interaction of donor-labeled SERCA2a with acceptor-labeled PLB or acceptor-labeled SERCA2a with donor-labeled PLB in membranes [12–14]. We previously showed that ophiopogonin D (OPD), a steroidal glycoside isolated from Radix Ophiopogonis (a tuber of Ophiopogon japonicas KerGawl), can induce the SERCA2a expression by upregulating the CYP2J3/EET system [12]. However, the relative contribution of CYP2J3 to OPD's effects on Ca^2+^ homeostasis and interaction between SERCA and PLB has not been investigated. Whether CYP2J3 participates in modulating the interaction between SERCA2a with PLB remains unknown.

Here, we report a compound extracted from the root of Radix Ophiopogonis as a potent CYP2J3 agonist. Our results demonstrate that OPD confers its anti-HF effects by inducing the CYP2J3 expression and subsequently promoting the interaction between SERCA2a and PLB, which is helpful for maintaining Ca^2+^ homeostasis.

## 2. Material and Methods

### 2.1. Antibodies and Reagents

OPD (purity: 98% by high-performance liquid chromatography) and isoproterenol·HCl (ISO) were purchased from the National Institutes for Control of Pharmaceutical and Biological Products (Beijing, China) and Sigma-Aldrich (St Louis, MO, USA), respectively. The TransScript™ First-Step RT-PCR SuperMixFast and SYBR® Green Master Mix were purchased from TransGen Biotech Co., Ltd. (Beijing, China). Antibodies against GAPDH and phosphorylated PLB (p-PLB) (Ser^16^/Thr^17^) were from Cell Signaling Technology Inc. (CST, Danvers, MA, USA). Antibodies against CaMKII, p-CaMKII, p-T197-PKA, PKA, p-PLB (p-Ser^16^), and SERCA2-ATPase were from Abcam (Cambridge, UK). The antibody against p-PLB (p-Thr1^7^) was from Zen BioScience (Chengdu, China) [15], and the antibody against CYP2J3 was from Bioss (Boston, MA, USA). The Universal Magnetic Co-IP Kit was obtained from Active Motif, Inc. (Carlsbad, CA, USA). Normal Rabbit IgG was from Cell Signaling Technology. Oregon Green 488 BAPTA was purchased from Invitrogen (Carlsbad, CA, USA). p-CMV-N-CFP-PLB, p-CMV-N-YFP-SERCA2a, and pLVX-IRES-ZsGreen-1-CYP2J3 were from Biomed (Beijing, China). The specific inhibitors of CaMKII (KN-93) and PKA (H-89) were purchased from Selleck Chemicals (Houston, TX, USA). Helicid was from TargetMol (Shanghai, China). All other reagents were purchased from commercial suppliers unless otherwise indicated.

### 2.2. Cell Culture and Treatment

H9c2 cells, a subclone of the original clonal cell line derived from embryonic BD1X rat heart tissue, were obtained from the American Type Culture Collection (Manassas, VA, USA). Cells were cultured in Dulbecco's Modified Eagle's Medium (DMEM) supplemented with 10% fetal bovine serum (FBS) and penicillin-streptomycin (100 IU/mL) in a humidified atmosphere of 95% air and 5% CO_2_ at 37°C. The cells were seeded in 96- or 6-cell culture plates for each experiment and grown in a humidified incubator containing 5% CO_2_. ISO was dissolved in distilled water. OPD, Helicid, H-89, and KN-93 were dissolved in dimethyl sulfoxide and diluted with DMEM.

### 2.3. Cell Viability Assay

Cell viability was determined with the Cell Counting Kit-8 (CCK-8) assay. Briefly, cells were seeded in 96-well plates at a density of 4 × 10^3^ per well in 100 mL complete medium. After being treated with different concentrations of OPD (1–80 *μ*mol/L), the cells were further incubated in a medium containing 0.5% CCK-8 for 0.5–4 h. The absorbance of 450 nm was measured with a microplate reader.

### 2.4. Measurement of ER-Free Ca^2+^

H9c2 cells were treated with different concentrations of OPD (0.25, 0.5, 1 *μ*mol/L) and ISO (1 *μ*mol/L). After 24 h, the ER Ca^2+^ concentration was measured using the Mag-Fluo-4/AM Kit from GENMED Scientific Inc. (Arlington, VA, USA), according to the supplier's instructions, as previously described [16, 17]. The cultured cells were incubated with 1 mL of 5 *μ*mol/L Mag-Fluo-4/AM for 30 min at 37°C to optimize loading into the ER. Cells were washed three times with D-Hank's solution to remove the probes. Relative ER Ca^2+^ was expressed as a percentage of the control.

### 2.5. Measurement of Ca^2+^-ATPase Activity

H9c2 cells were treated with OPD and ISO as previously mentioned. The enzyme activity of SERCA2a in SR was detected with a microplate system. SERCA2a vesicles were obtained as previously described for the measurement of its activity [18], which was detected using an inorganic phosphorus colorimetric method with the Ultramicro-ATPase Assay Kit (Jiancheng, China). Briefly, this test includes an enzymatic and phosphorus reaction based on the theory that ATP is decomposed into ADP and inorganic phosphate (Pi) by ATPase. The activity of ATPase was measured by the quantity of Pi, and the amount of Pi decomposed by ATPase per mg tissue or cell protein per h (*μ*mol Pi/mg prot/h) was regarded as one unit of the ATPase activity. The values for the maximal SERCA2a activity were taken directly from the experimental data and normalized for total protein concentration (mmol/g protein/min). These data were analyzed by nonlinear regression with computer software (GraphPad Software, San Diego, CA, USA).

### 2.6. RNA Isolation and Quantitative PCR

After being treated with the corresponding drugs as described above, total RNA was extracted. The expression of specific mRNAs was quantified with quantitative PCR (qPCR) as previously described. Briefly, total RNA was prepared from H9c2 cells using Trizol reagent. The RNA integrity and concentration were measured using the DU-600 ultraviolet spectrophotometer (Beckman Coulter, Brea, CA, USA). All RNA samples with an OD260/OD280 between 1.8 and 2.0 were used for qPCR. Total RNA was reverse transcribed with the Transcriptor First-strand cDNA Synthesis Kit to obtain the cDNAs. qPCR was performed using the ABI Prism 7500 Real-Time PCR System (Applied Biosystems, Waltham, MA, USA) using the Trans Script™ SYBR® Green Master Mix Kit, according to the instructions provided. Each target gene was quantified, with GAPDH serving as the internal control. The primer sequences are listed in Table 1.

### 2.7. Western Blot Analysis

Protein concentrations were determined using a BCA protein assay kit. Proteins were analyzed by sodium dodecyl sulfate-polyacylamide gel electrophoresis (SDS-PAGE) and blotted using standard protocols [19]. The expression was quantified by densitometry and normalized to the GAPDH expression. Then, all groups were normalized to their respective controls.

### 2.8. Coimmunoprecipitation Assay

A coimmunoprecipitation (co-IP) assay was conducted to examine the physical association between SERCA2a and p-PLB/PLB. A co-IP assay was performed using the Universal Magnetic Co-IP Kit (Active Motif) according to the manufacturers' protocol. Briefly, H9c2 cells were treated with phosphate-buffered saline (PBS)/inhibitor solution and collected by brief centrifugation and lysed in lysis buffer. Protein complexes were immunoprecipitated using SERCA2a antibody (CST) or IgG as a control. The immunoprecipitation was performed overnight at 4°C. The complexes were incubated with Protein G Magnetic Beads for 1 h at 4°C on a rotator. The resin was washed four times with 500 *μ*L complete co-IP/wash buffer. The eluted protein complexes were resolved by SDS-PAGE (10% gel) and detected with PLB/p-PLB antibody.

### 2.9. Immunofluorescence Colocalization

The colocalization of SERCA2a and PLB/p-PLB proteins in ISO-induced HF was detected. H9c2 cells were cultured in DMEM supplemented with 10% FBS and penicillin-streptomycin (100 IU/mL) in a humidified atmosphere of 95% air and 5% CO_2_ at 37°C. The cells were seeded on laser confocal small dish for each experiment and grown in a 37°C humidified incubator containing 5% CO_2_. After 24 h drug treatment, the cell culture media was removed, and cells were washed with 1× PBS. The cells were fixed in 100% ice-cold methanol for 10–15 min at room temperature and allowed to dry for 15 min. Next, the cells were washed twice with 1× PBS/IFA for 5 min and permeabilized with 0.3% Triton-X 100 in 1× PBS/IFA for 10 min followed by washing twice with 0.03% Triton-X 100 for 5 min. The cells were blocked in 5% BSA in 1× PBS/IFA for 1 h. Then, the cells were incubated with the appropriate primary antibody (rabbit-SERCA2a, mouse-PLB, rabbit-p-PLB; both diluted 1 : 1000) at 4°C for overnight. After washing, the cells were incubated with the corresponding secondary antibody (goat anti-mouse IgG, FITC-conjugated and goat anti-rabbit IgG, RBITC-conjugated), diluted 1 : 500 for another 1 h. 4′6-diamidino-2-phenyllindole (DAPI) was added to cells at a dilution of 1 : 2000 for 1 h in the dark. Then, the cells were washed six times with 0.03% Triton-X 100 for 5 min and sealed with the Prolong® Gold antifade reagent. Sections were examined using the LSM800 confocal laser scanning microscope. The immersion-oil Plan Neofluar 60/0.75 objective was used. Double fluorescence for green and red channels was imaged using excitation of an argon-krypton laser at wavelengths of 488 and 543 nm. Images were acquired and processed for colocalization analysis in the TIFF format. Quantitative analysis was performed employing ImageJ Pro Plus Software.

### 2.10. H9c2 Cell Culture and Small Interfering RNA Transfection

H9c2 cells were seeded at a density of 1.2 × 10^6^/well in a 6-well dish. At 12 h after plating, the cells were incubated with 80 nmol/L CYP2J3 small interfering RNA (siRNA) and control siRNA in 1 mL Opti-MEM medium for 6 h as previously described. After 6 h, new serum-free DMEM with OPD and ISO was added. Cells were incubated for another 24 or 48 h for different experiments.

### 2.11. Small-Molecule Inhibition of Kinase and Metabolic Targets

H-89 and KN-93 (1 *μ*mol/L; Selleck), specific inhibitors for PKA and CaMKII, respectively, were used to investigate PKA/Ser^16^-PLB, and CaMKII/Thr^17^-PLB signaling pathways mediated the effect of OPD. Cells were treated with the abovementioned medium containing H-89 and KN-93 for 6 h, finally cultured in a medium containing ISO (1 *μ*mol/L) and the highest concentration of OPD (1 *μ*mol/L).

### 2.12. Molecular Biology and HEK293T Cell Culture

Rat PLB fused to the C-terminus of the cyan fluorescent protein (CFP), and rat SERCA2a fused to the C-terminus of the enhanced yellow fluorescent protein (YFP). Extraction of fluorescent protein plasmid was performed with Qiagen Company's Plasmid Extraction Kit (Plasmid DNA Maxiprep Kit; Germantown, MD, USA). HEK293T cells were cultured on dishes coated with polylysine. The dishes were filled with complete DMEM growth medium containing 10% heat-inactivated FBS. The cells were transfected according to the manufacturer's instructions after the cell density reached 10%. MEM growth medium was used instead of complete DMEM growth medium during the 6 h incubation of HEK293T with plasmid. For the FRET experiments, HEK293T cells were cotransfected with CFP-PLB plasmid and YFP-SERCA2a plasmid at a concentration of 500 ng/*μ*L.

### 2.13. Plasmid Detection and Time-Resolved FRET Imaging

Plasmids were transfected into HEK293T cells according to the abovementioned procedures. The DNA sequencing results of the two recombinant plasmids were correct and can be used in downstream experiments. To verify the transfection efficacy of CFP-PLB plasmid and YFP-SERCA2a PLB into HEK293T cells, after a 24 h transfection, cells were lysed, and proteins from the cell lysates were resolved by electrophoresis followed by western blot analysis (see Section 2.7). The mixture was incubated with horseradish peroxidase-conjugated goat-anti-mouse or goat-anti-rabbit IgG secondary antibodies (1 : 2000) for 1 h at room temperature and visualized using the ImageQuant LAS 500 (Healthcare Bio-Sciences AB, Uppsala, Sweden). Fluorescence imaging was performed with an inverted microscope equipped with the Plan-Apochromat 100×/1.40 Oil DIC M27 objective. For epifluorescence imaging, illumination was introduced through an excitation filter wheel equipped with 427/10 nm (for CFP) and 504/12 nm (for YFP) narrowband filters and a multiple band dichroic mirror. Emission was detected with a back-thinned electron-multiplying charged couple device camera (LSM 880; AxioObeserver, Zeiss, Oberkochen, Germany) through the emission filter wheel, 472/30 nm (for CFP) and 542/27 nm (for YFP). “Prismless” total internal reflection fluorescence (TIRF) used the 458-nm Ar laser line, directed through the objective with a multiple band dichroic mirror. TIRF emission was selected with filters described above. For spatially resolved, acceptor-selective photobleaching, the Ar laser 514-nm line was selected by a laser line filter and directed to the sample with a10/90 beam splitter. The bleach beam was focused to a spot on the specimen using a Keplerian telescope composed of two planoconvex lenses. Laser photobleaching exposure time was controlled by a Uniblitz shutter and was typically for 54.55 s with 20 frames. The above activity was measured using software (ZEN). Image records from multiple experiments were averaged together to reduce error. Analysis of the evolution of the line-out profiles was performed by fitting image data with a custom analysis program written in Graphpad Software.

### 2.14. Statistical Analyses

All data were expressed as the mean ± standard deviation (SD). Comparisons between groups were performed by a one-way analysis of variance. *P* < 0.05 was considered statistically significant.

## 3. Results

### 3.1. Effects of OPD on the Viability of H9c2 Cells

**Figure 1:**
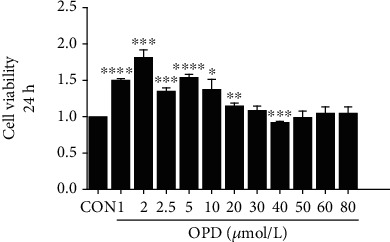
Effect of OPD on the viability of H9c2 cells. H9c2s were treated with different concentrations of OPD (1–80 *μ*mol/L) for 24 h, and the cell viability was detected by the CCK-8 assay, ^∗^*P* < 0.05, ^∗∗^*P* < 0.01, and ^∗∗∗^*P* < 0.005 versus the control group, *n* = 4 per group.

Cells were treated with different concentrations of OPD (1–80 *μ*mol/L), and the viability of H9c2 cells was detected by the CCK-8 assay. The cell viability was apparently decreased after the incubation with OPD. The results suggested that the responses stimulated by OPD on cell viability were concentration-dependent. High concentrations of OPD had obvious inhibitory effects on cell vitality. To investigate the effects of OPD on cardiomyocytes, OPD concentrations of 0.25, 0.5, and 1 *μ*mol/L were used in subsequent experiments (Figure 1).

### 3.2. Activation of the CYP2J3 Expression in H9c2 Cells and HEK293T Cells by OPD

**Figure 2:**
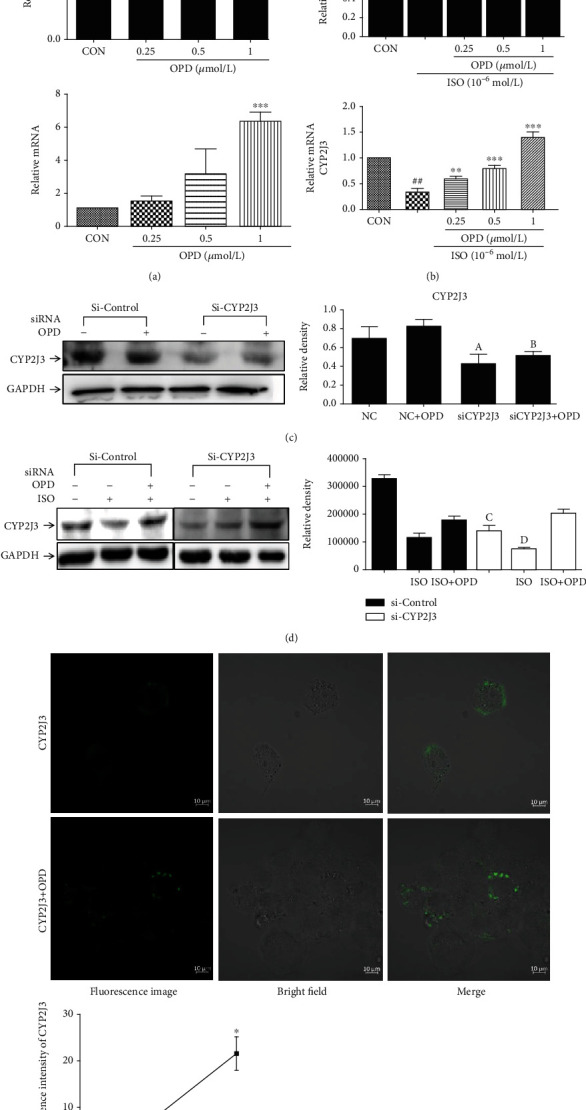
Effect of OPD on the expression of CYP2J3 in H9c2 cells and 293 T cells. (a) H9c2s were treated with different concentrations of OPD (0.25, 0.5, 1 *μ*mol/L) for 24 h. CYP2J3 mRNA and protein expression were increased in OPD-treatment groups ^∗^*P* < 0.05, ^∗∗^*P* < 0.01 versus the control group; ^∗∗∗^*P* < 0.005 versus the ISO-treatment group (1 *μ*mol/L). (b) H9c2s were cotreated with ISO (1 *μ*mol/L) and different concentrations of OPD (0.25, 0.5, 1 *μ*mol/L) for 24 h. CYP2J3 mRNA and protein expression were decreased in the ISO-treatment group ^###^*P* < 0.005 versus the control group; ^∗^*P* < 0.05, ^∗∗^*P* < 0.01, and ^∗∗∗^*P* < 0.005 versus the ISO-treatment group. (c) CYP2J3-siRNA and negative control- (NC-) siRNA were transfected into H9c2s. Cells were treated with OPD (1 *μ*mol/L) and ISO (1 *μ*mol/L) as mentioned above. CYP2J3 mRNA and protein expression were decreased in the Si-CYP2J3 group, ^a^*P* < 0.05 versus the si-control group; the CYP2J3 protein and mRNA level were increased in the OPD-treated group, ^b^*P* < 0.01 versus the OPD-treated NC group. (d) CYP2J3-siRNA and NC-siRNA were transfected with H9c2s. Cells were cotreated with OPD (1 *μ*mol/L) and ISO (1 *μ*mol/L) as mentioned above. CYP2J3 mRNA and protein expression were decreased in the si-CYP2J3 group, ^c^*P* < 0.05 versus the si-control group; CYP2J3 mRNA and the protein expression were decreased in the ISO-treated si-CYP2J3 group, ^d^*P* < 0.01 versus the ISO-treated NC group. (e) 293 T cells were transfected with pLVX-IRES-ZsGreen-1-CYP2J3 plasmid for 24 h. Cells were treated with OPD (1 *μ*mol/L) for 24 h. The CYP2J3 expression was increased in the OPD-treated+CYP2J3 transfected group versus the CYP2J3 transfected group, *n* = 3 per group.

To determine the effect of OPD on the CYP2J3 protein expression in cardiomyocytes, we performed immunoblot analyses and qPCR. OPD qualitatively induced the CYP2J3 mRNA and protein expression as expected (Figure 2(a)), in accordance with previous results [16, 20]. Next, we determined the effects of OPD on CYP2J3 using an ISO-induced cell model, which represents a state of heart failure. H9c2 cell lysates were prepared for the estimation of the CYP2J3 immunoreactive protein; GAPDH was used to indicate protein loading. From Figure 2(b), the CYP2J3 mRNA and protein expression was increased at each concentration by OPD and ISO cotreatment. ISO is a *β*-adrenergic receptor agonist, which induces HF both in vitro and in vivo [21]. The expression of CYP2J3 was decreased in the ISO-induced HF group. The role of OPD was illustrated by data showing that transfection of CYP2J3 siRNA into H9c2 cells, which inhibited the CYP2J3 expression, abrogated the inhibitory of ISO on CYP2J3 degradation (Figures 2(c) and 2(d)). pLVX-IRES-ZsGreen-1-CYP2J3 plasmid was transfected into HEK293T cells for 24 h, and cells were treated with OPD for another 24 h. The fluorescence intensity of CYP2J3 was detected by laser confocal focusing (Figure 2(e)). Compared with the CYP2J3 group, the fluorescence intensity in the OPD-treatment group was significantly increased.

### 3.3. OPD Induces Colocalization of SERCA2a and p-PLB through CYP2J3 at the ER

**Figure 3:**
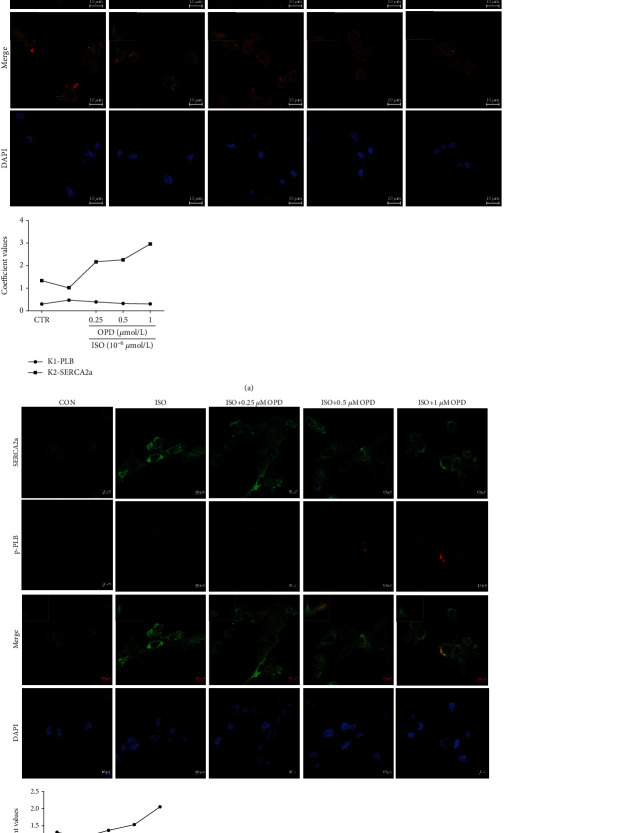
OPD induces SERCA2a colocalization of SERCA2a and p-PLB in HF cells. Images were collected on the LSM880 confocal microscope with Plan-Apochromat ×63. (a) Images showing immunofluorescence of SERCA2a (red) and PLB (green) proteins in H9c2 cells and changes of their colocalization pattern following OPD (1 *μ*mol/L) and ISO (1 *μ*mol/L) administration. An embedded scattergram estimated the amount of each detected antigen based on colocalization. Colocalized pixels of yellow color are located along the diagonal of the scattergram. Compared with the control group, the green (PLB) intensity increased in the ISO-treated group while the red (SERCA2a) intensity decreased. The OPD-treated group showed decreased PLB intensity compared with the ISO-treated group.(b) Images showing immunofluorescence of SERCA2a (green) and p-PLB (red) proteins in H9c2 cells and changes of their colocalization pattern following OPD (1 *μ*mol/L) and ISO (1 *μ*mol/L) administration. An embedded scattergram estimated the amount of each detected antigen based on colocalization. Colocalized pixels of yellow color are located along the diagonal of the scattergram. Compared with the control group, the red (p-PLB) intensity decreased in the ISO-treated group. The OPD-treated group showed increased p-PLB intensity compared with the ISO-treated group.

**Figure 4:**
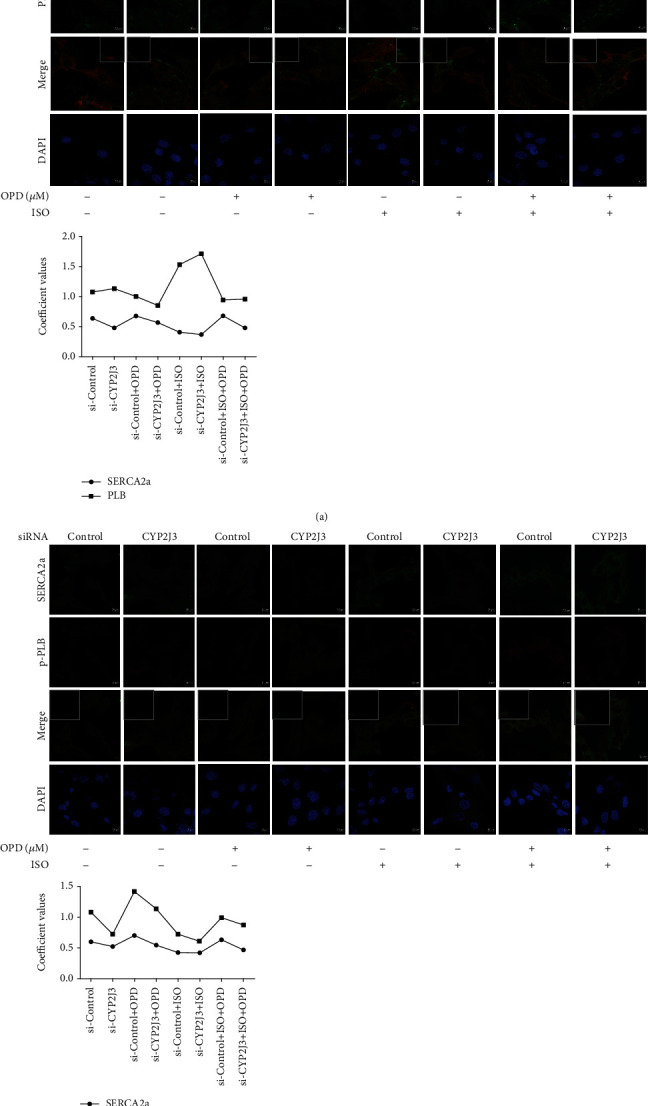
OPD induced SERCA2a colocalization of SERCA2a and p-PLB through CYP2J3 in H9c2 cells. Cells were transfected with CYP2J3 siRNA for 24 h. ISO and OPD were administrated as mentioned above. (a) Images showing immunofluorescence of SERCA2a (red) and PLB (green) proteins in H9c2 cells and changes of their colocalization. An embedded scattergram estimated the amount of each detected antigen based on colocalization. Colocalized pixels of yellow color are located along the diagonal of the scattergram. Compared with the NC group, the green (PLB) intensity increased in the Si-CYP2J3 group while the red (SERCA2a) intensity decreased. The OPD-treated si-CYP2J3 group showed decreased PLB intensity compared with the OPD-treated NC group. (b) Images showing immunofluorescence of SERCA2a (green) and p-PLB (red) proteins in H9c2 cells and changes of their colocalization pattern following OPD (1 *μ*mol/L) and ISO (1 *μ*mol/L) administration as mentioned above. An embedded scattergram estimated the amount of each detected antigen based on colocalization. Colocalized pixels of yellow color are located along the diagonal of scattergram. Compared with the NC group, the red (p-PLB) intensity decreased in the si-CYP2J3 group. The OPD-treated si-CYP2J3 group showed increased p-PLB intensity compared with the OPD-treated NC group.

To determine whether SERCA2a colocalized with PLB/p-PLB, colocalization of three proteins in H9c2 cells was examined by immunofluorescence using a confocal microscope. The results showed some colocalization between SERCA2a and PLB/p-PLB to different degrees. OPD induced colocalization between SERCA2a and p-PLB (Figure 3(b)), while SERCA2a and PLB protein showed decreased colocalization in H9c2 cells after OPD treatment (Figure 3(a)). Transfection of CYP2J3 siRNA was performed to test the CYP2J3 function in the colocalization of SERCA2a/PLB and SERCA2a/p-PLB. After the transfection of CYP2J3 siRNA, H9c2 cells were treated with OPD and ISO as mentioned above to determine the induction of OPD in HF cells (Figures 4(a) and 4(b)). Images show immunofluorescence of SERCA2a (green) and p-PLB (red) proteins in H9c2 cells and changes of their colocalization pattern following the OPD (1 *μ*mol/L) and ISO (1 *μ*mol/L) administration as mentioned above. An embedded scattergram estimated the amount of each detected antigen based on colocalization. Colocalized pixels of yellow color are located along the diagonal of the scattergram. Compared with the NC group, the red (p-PLB) intensity decreased in the si-CYP2J3 group. The OPD-treated si-CYP2J3 group showed increased p-PLB intensity compared with the OPD-treated NC group.

### 3.4. OPD Increases the Physical Association between SERCA2a and p-PLB through CYP2J3

**Figure 5:**
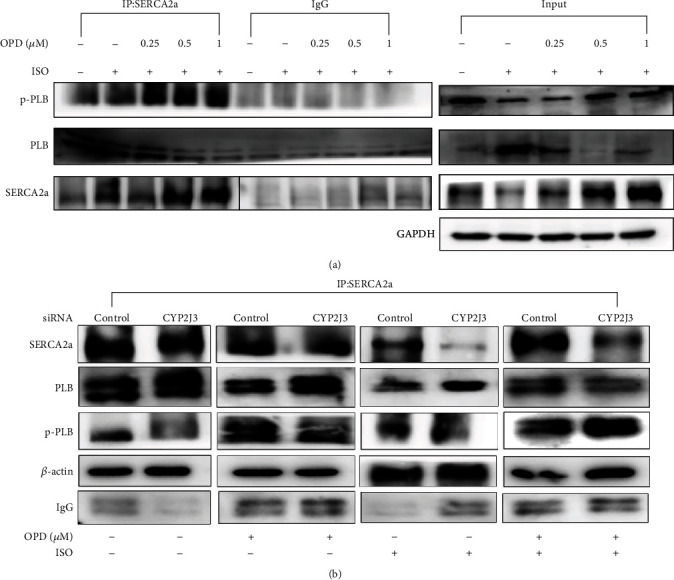
SERCA2a co-IP analysis of p-PLB and PLB proteins. (a) H9c2 cells were pretreated with ISO (1 *μ*mol/L) and OPD (0.25, 0.5, 1 *μ*mol/L) at the indicated concentrations mentioned above. The physical association between SERCA2a and PLB/p-PLB was examined by a co-IP assay. Compared with the ISO-treated group, OPD induced the interaction between SERCA2a and p-PLB in a dose-dependent manner. (b) CYP2J3-siRNA and NC-siRNA were transfected into H9c2s. Cells were cotreated with OPD (1 *μ*mol/L) and ISO (1 *μ*mol/L) as mentioned above. Western blot analysis of the interaction between SERCA2a and p-PLB/PLB proteins by co-IP. CYP2J3 depletion weakened the physical binding of SERCA2a and p-PLB in H9c2 cells, and OPD treatment improved the faint binding caused by CYP2J3.

To evaluate the influence of OPD in the interaction of p-PLB with SERCA2a, H9c2 cells were treated with OPD and ISO as mentioned above. Total cellular proteins were extracted. After preclearing with protein G sepharose beads, the cell lysates were incubated with or without an anti-SERCA2a antibody followed by incubation with protein G sepharose beads. The co-IP'd protein complexes were eluted and analyzed by western blotting using antibodies against p-PLB and PLB. Co-IP'd bands were seen for p-PLB and PLB (Figure 5(a)), while the antibody used could detect the capsid protein in western blot analysis. The co-IP assay demonstrated that the endogenous SERCA2a protein was specifically co-IP'd with p-PLB in an OPD-dependent manner (Figure 5(a)). To confirm the regulatory function of CYP2J3 in the interaction between SERCA2a and p-PLB/PLB proteins, H9c2 cells were transfected with CYP2J3 si-RNA for 24 h, and the immunoprecipitations were repeated. Membranes were initially probed with an antibody against SERCA2a and subsequently with antibodies against the immunoprecipitating protein. The results suggest that the suppression of CYP2J3 alleviates the physical binding between SERCA2a and p-PLB (Figure 5(b)). Furthermore, OPD may induce the expression of CYP2J3 to promote the interaction of SERCA2a and p-PLB.

### 3.5. OPD Increases the Ca^2+^-ATPase Activity and Ca^2+^ Uptake in the ER after 24 H of ISO Supplementation

**Figure 6:**
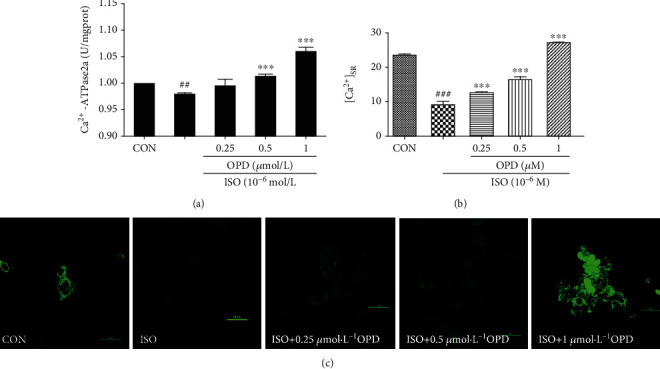
Effects of OPD on ER Ca^2+^ uptake and Ca^2+^-ATPase activity in HF cells. (a) Effects of OPD on the Ca^2+^-ATPase activity measured by the quantity of Pi. The activity of SERCA2a was decreased in the ISO-treatment group, ^##^*P* < 0.01 versus the control group; OPD (1 *μ*mol/L) induced the Ca^2+^-ATPase activity, ^∗∗∗^*P* < 0.005 versus the ISO-treatment group. (b, c) Effects of OPD followed ISO (1 *μ*M) on [Ca^2+^]_SR_. Application of 1 *μ*M ISO indicated complete depletion of [Ca^2+^] in the SR compared with the control group, ^###^*P* < 0.005. Twenty-four hour application of OPD increased the Ca^2+^ uptake in ER, ^∗∗∗^*P* < 0.005 versus the ISO-treated group, *n* = 3 per group.

ISO supported Ca^2+^ uptake in the ER was significantly lower than the control. In addition, compared with the ISO-treated group, OPD supported Ca^2+^ uptake was much higher (Figures 6(b) and 6(c)). Next, we explored the possible action of OPD on the Ca^2+^-ATPase. The activity of Ca^2+^-ATPase was weaker in ISO-treated HF cells than in nontreated control cardiomyocytes. Importantly, treatment with OPD markedly improved the activity of Ca^2+^-ATPase in a dose-dependent manner (Figure 6(a)).

### 3.6. OPD Activates PLB Phosphorylation in HF Cells

**Figure 7:**
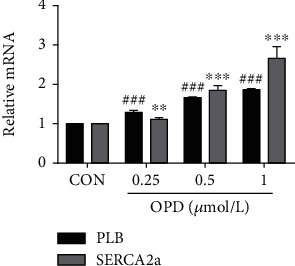
Effects of OPD on the expression of SERCA2a and PLB mRNA. The PLB and SERCA2a mRNA expression increased compared with the control group, ^∗∗^*P* < 0.01, ^∗∗∗^*P* < 0.005, ^###^*P* < 0.005, *n* = 3 per group.

**Figure 8:**
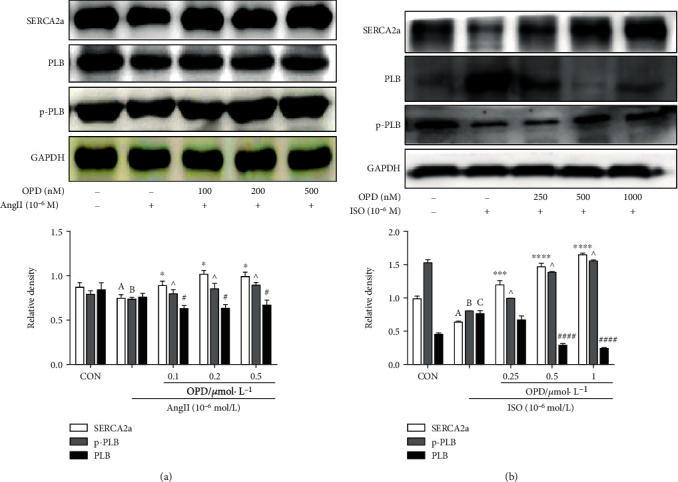
Effects of OPD on the phosphorylated status of PLB in different HF cardiomyocytes. (a) SERCA2a and p-PLB protein levels were decreased in AngII-induced HF cells, ^a,b^*P* < 0.005 versus the control group. The PLB expression increased compared with the control group, ^c^*P* < 0.005. Different concentrations of OPD (0.1, 0.2, 0.5 *μ*mol/L) reversed the change compared with the AngII-treated group, ^∗^*P* < 0.05, ^^^*P* < 0.05, ^#^*P* < 0.05. (b) SERCA2a and p-PLB protein levels were decreased in ISO-induced HF cells, ^a,b^*P* < 0.005 versus the control group. The PLB expression increased compared with the control group, ^c^*P* < 0.005. Different concentrations of OPD (0.25, 0.5, 1 *μ*mol/L) reversed the change compared with the ISO-treated group, ^∗^*P* < 0.05, ^^^*P* < 0.05, ^#^*P* < 0.05, *n* = 3 per group.

To verify whether OPD transcriptionally modulates the expression of proteins involved in Ca^2+^ transportation, we performed qPCR to measure the steady-state levels of mRNA encoding the subunits of SERCA2a and PLB. As already observed at the protein level, the expression of SERCA2a and PLB mRNA was increased in the OPD-treated group compared with the controls (Figure 7). The phosphorylation status of PLB was able to remove the inhibitory effect of PLB on SERCA2a, resulting in increased SR-Ca^2+^ uptake and enhanced myocyte contractility and relaxation (Ca^2+^ and excitation-contraction coupling in the heart). We actually measured a marked decrease in total PLB associated with a more evident increase in the phosphorylated form of the protein, resulting in a significant increase in the p-PLB/PLB ratio. Consistent with this finding, a more efficient intracellular Ca^2+^ dynamic was observed in H9c2 cells. It is worth noting that OPD-treated cells exhibited a significantly higher p-PLB/PLB ratio compared with ISO-induced (Figure 8(a)) and AngII-induced (Figure 8(b)) HF cardiomyocytes.

### 3.7. Inhibition of the PKA and CaMKII Cascade Does Not Abolish the Effect of OPD on Phosphorylation of PLB

**Figure 9:**
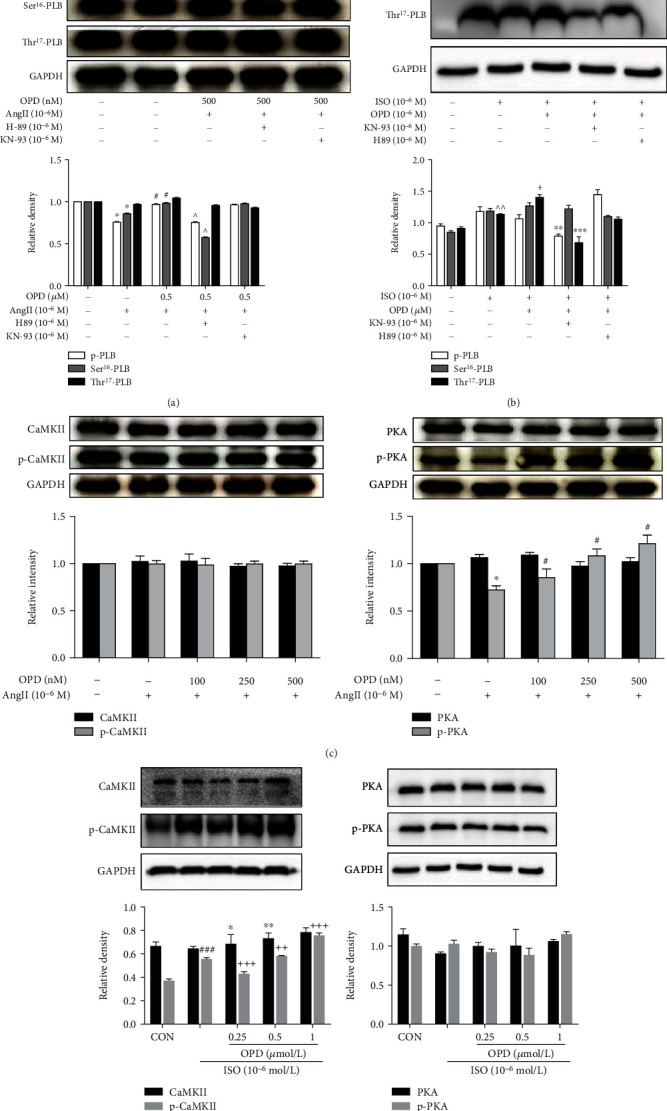
Effects of OPD on the phosphorylated status of PLB in ISO or Ang-II induced HF cells. (a) H-89 selectively inhibited Ser^16^ phosphorylation of PLB during AngII and OPD cotreatment. (b) KN-93 selectively inhibited Thr^17^ phosphorylation of PLB during ISO (1 *μ*mol/L) and OPD (1 *μ*mol/L) cotreatment. (c) PKA was phosphorylated in Ang-II induced HF cells, ^∗^*P* < 0.05 versus the control group. OPD promoted the expression of p-PKA (the activation state of PKA), ^#^*P* < 0.05 versus the Ang-II-treated group. Furthermore, there was no significance in the expression of CaMKII and p-CaMKII. (d) CaMKII was phosphorylated in ISO-induced HF cells, ^###^*P* < 0.005 versus the control group. OPD promoted the expression of p-CaMKII (activation state of CaMKII), ^++^*P* < 0.05, ^+++^*P* < 0.05 versus the ISO-treated group, *n* = 3 per group. Furthermore, there was no significance in the expression of PKA and p-PKA.

It is already known that PLB is phosphorylated by either PKA at the Ser^16^ residue or CaMKII at the Thr^17^ residue [22]. To examine whether activation of the two kinase pathways by OPD results in the phosphorylation of PLB-mediated regulation of the SERCA2a, we performed a series of experiments with chemical modulators of the PKA and CaMKII pathway, such as the H-89 (PKA inhibitor) and KN-93(CaMKII inhibitor). The results showed a clear dissociation between the phosphorylation of Ser^16^ and Thr^17^ residues of PLB after AngII and ISO treatment, respectively. The H-89 (PKA inhibitor) selectively induced a decrease in Ser^16^ phosphorylation during AngII and OPD cotreatment (Figure 9(a)). In other words, OPD played a role in the phosphorylation of PLB-Ser^16^ through the AngII-induced PKA cascade. As a result, we examined the expression of PKA and p-PKA after AngII and OPD cotreatment. We found that OPD induced the activation of PKA cascade in AngII-treated HF cells (Figure 9(c)). Interestingly, CaMKII seems not to be activated in the AngII-induced HF cell model (Figure 9(c)). During ISO treatment, KN-93 (CaMKII) selectively induced a decrease in Thr^17^ phosphorylation after OPD application (Figure 9(b)). OPD selectively induced phosphorylation of PLB-Thr^17^ through the CaMKII pathway with ISO treatment in H9c2 cells. OPD induced the expression of phosphorylated CaMKII in ISO-treated HF cells (Figure 9(d)), indicating that selective underlying mechanisms are involved in the phosphorylation of two sites. This point was further explored in subsequent experiments.

### 3.8. The FRET Assay of HEK293T Cells Expressing SERCA2a and PLB

**Figure 10:**
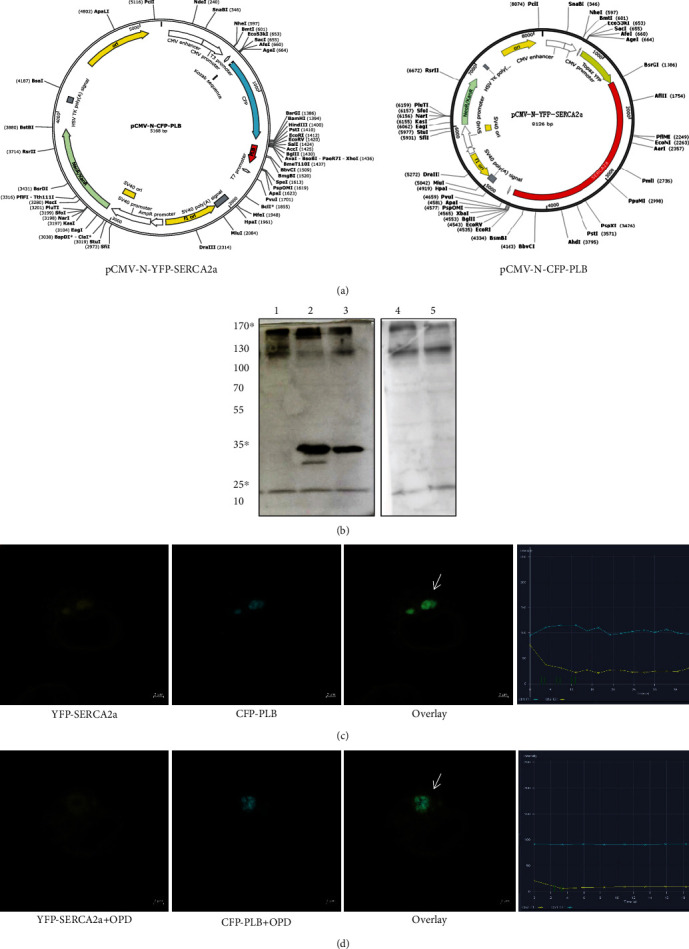
The FRET assay of HEK293T cells expressing SERCA2a and PLB. (a) The plasmid profiles of pCMV-N-YFP-SERCA2a and pCMV-N-CFP-PLB. (b) Immunoblot of CFP-PLB, YFP-SERCA2a, and CFP/YFP with anti-PLB monoclonal antibody, anti-SERCA2a antibody, and anti-GFP antibody, respectively. Lanes from the left are (1) pCMV-N-YFP-SERCA2a (predicted band size: 170 kDa), (2) pCMV-N-CFP-PLB (predicted band size: 35 kDa), (3) pCMV-N-CFP-PLB+pCMV-N-YFP-SERCA2a, (4) untransfected HEK293T cell homogenate control, and (5) pCMV-N-YFP + pCMV-N-CFP (predicted band size: 27 kDa). (c) Profiles of YFP-SERCA2a (yellow points) and CFP-PLB (blue points) fluorescence at 10 s after YFP-selective laser spot photobleaching. The data are described in the line chart, and the arrow shows the relationship between YFP-SERCA2a and CFP-PLB fluorescence across the target region. The intensity indicates 18% energy transfer. (d) Profiles of YFP-SERCA2a (yellow points) and CFP-PLB (blue points) fluorescence after OPD treatment. There is no obvious change in the CFP intensity after YFP-selective laser spot photobleaching.

To optimize the conditions of transient transfection in HEK293T cells, cells were infected with YFP-SERCA2a and CFP-PLB (Figure 10(a)) for 24 h. Total cellular proteins were extracted, resolved by electrophoresis, and transferred to nitrocellulose membranes before being subjected to western blot analysis to determine the expression of PLB and SERCA2a with GAPDH used as a loading control. The results showed the expected band sizes of SERCA2a (170 kDa), PLB (35 kDa), and GAPDH (25 kDa), which indicated the suitable transfection conditions (Figure 10(b)). When two fluorescent probes are brought into close proximity (<100 Å), they can undergo FRET [23, 24]. FRET between YFP-SERCA2a and CFP-PLB was detected by selective photobleaching of YFP. CFP fluorescence increased locally after brief YFP exposure to a focused spot of 514 nm laser illumination (Figure 10(c), arrow). CFP-SERCA intensity (postbleach/prebleach) indicated that about 18% increase in CFP-SERCA fluorescence was restricted to the target site (Figure 10(c)). Donor enhancement after acceptor photobleaching is diagnostic of FRET [25]. After OPD pretreatment for 6 h, FRET between YFP-SERCA2a and CFP-PLB was observed as mentioned above (Figure 10(d), arrow). The intensity of CFP did not significantly change after YFP photobleaching (Figure 10(d)). This may be attributed to OPD blocking the unphosphorylated status of PLB.

### 3.9. OPD Induces Pregnane X Receptor Nuclear Translocation

**Figure 11:**
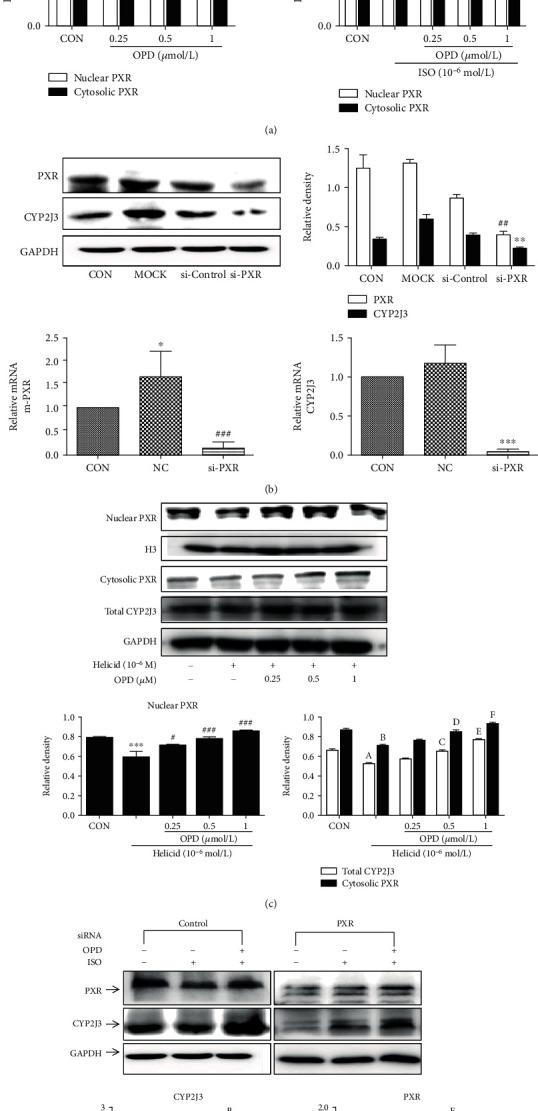
The expression of CYP2J3 decreased after the PXR was knocked down. (a) OPD induces PXR nuclear translocation in H9c2 cells. ^∗^*P* < 0.05, ^∗∗^*P* < 0.01 compared with the control group. (b) The expression of CYP2J3 decreased after the PXR was knocked down, ^∗∗∗^*P* < 0.001, ^###^*P* < 0.001 versus the NC group. The effect of OPD on the transcription of the genes encoding CYP2J3 in HF H9c2 cells transfected with siPXR. ^a^*P* < 0.01, ^d^*P* < 0.01 versus the si-control group. ^b^*P* < 0.05, ^c^*P* < 0.05 versus the si-control group. ^e^*P* < 0.001, ^f^*P* < 0.001 versus the si-control group. (c) The expression of CYP2J3 decreased after the PXR was knocked down.^∗∗∗^*P* < 0.001 versus the control group, ^#^*P* < 0.05, ^###^*P* < 0.001 versus the Helicid group. ^a^*P*<0.05, ^b^*P*<0.01 versus the control group. ^c^*P*<0.05, ^d^*P*<0.05, ^e^*P*<0.05, ^f^*P* < 0.05 compared with the Helicid group. (d) The effect of OPD on the expression of CYP2J3 and PXR after PXR was knocked down in HF H9c2 cells. ^a^*P* < 0.01, ^d^*P* < 0.01 versus the si-control group. ^b^*P* < 0.05, ^c^*P* < 0.05 versus the si-control group. ^e^*P* < 0.001, ^f^*P* < 0.001 versus the si-control group, *n* = 3 per group.

It is known that pregnane X receptor (PXR) is a “master regulator” or “xenosensor” for the metabolism and clearance of diverse endogenous and exogenous compounds [26]. When a ligand (prescription drugs, herbal supplements, vitamins, and other endobiotics) binds to PXR [22], target genes are upregulated through transcriptional induction. CYP3A4 is the most important and well-studied target gene of PXR, which is the crucial drug/xenobiotic enzyme and metabolizes about 50% of all drugs and xenobiotics [27]. In addition, PXR regulates the expression of several other important drug/xenobiotic-metabolizing enzymes such as CYP2C9, CYP2B6, CYP2C19, and CYP3A5 [22]. The regulation of CYP2J is relatively deficient when compared with other isoforms belonging to the CYP2 subfamily, such as CYP2C, 2D, and 2E. Furthermore, the transcriptional regulator of CYP2J3 still remains ambiguous. Our previous study demonstrated that OPD promoted the translocation of PXR from the cytoplasm to the nucleus in a concentration-dependent manner (Figure 11(a)). To identify the regulatory effect of PXR on CYP2J3, the expression of CYP2J3 decreased after the transfection of PXR-siRNA into H9c2 cells (Figure 11(b)). In addition, OPD reversed the decline in the PXR expression after Helicid (specific inhibitor of PXR) treatment (Figure 11(c)). After the transfection of si-PXR in ISO-induced HF H9c2 cells, the expression of CYP2J3 decreased, and OPD could reverse this decrease (Figure 11(d)).

## 4. Discussion and Conclusions

OPD is a steroidal glycoside isolated from Radix ophipogomis, which has recently garnered increasing interest for its anti-inflammatory and antioxidative activities in different cardiovascular diseases [28–30]. Our previous studies first reported that OPD can induce both the CYP2J3 and CYP2J2 expression. Specifically, OPD significantly reduced intracellular Ca^2+^ overload by upregulating the levels of CYP2J3/EETs in rat myocardial cells. Moreover, OPD promoted Ca^2+^ homeostasis by regulating Ca^2+^ handling proteins, which included SERCA2a and PLB [29]. The above studies in our laboratory reported for the first time that OPD can effectively induce CYP2J and affect the expression of calmodulin. However, there is no in-depth study on whether OPD can directly affect the interaction between SERCA2a and PLB, or whether CYP2J3 participates in modulating the interaction between SERCA2a with PLB remains unknown. The current study is a further study of the above work. It is first confirmed that OPD can directly enhance the interaction between SERCA and phosphorylated PLB. Our previous two in vivo studies verified the cardiovascular protective effect of OPD through the ischemia-reperfusion model and myocardial hypertrophy animal model [20]. In the current study, we reported that in vitro OPD can exert its anti-HF effects by inducing the interaction between SERCA2a and PLB through CYP2J3, identifying CYP2J3 as a direct intracellular target of OPD and providing important insight into the mechanism of OPD actions. The primary myocardial cells, as the cells derived from the myocardial tissue, are closest to the myocardial tissue. In the further study, we will verify the effect of OPD on HF in mice such as the TCA heart failure model or primary myocardial cells. The previous experiment results in our lab demonstrated that OPD (0.1 *μ*mol/L ~80 *μ*mol/L) had no obvious toxic effect on cells (cell viability > 84%). Besides, cells were treated with different times and concentrations of OP-D (0.1~200 *μ*mol/L), the viability of HUVECs was detected by MTT assay, and the results suggested that high concentrations (80 *μ*mol/L) of OPD had obvious inhibitory effect on cell vitality (>85%) [20]. Similarly, in the current experiment, OPD had no obvious toxic effect on H9c2 cells.

**Figure 12:**
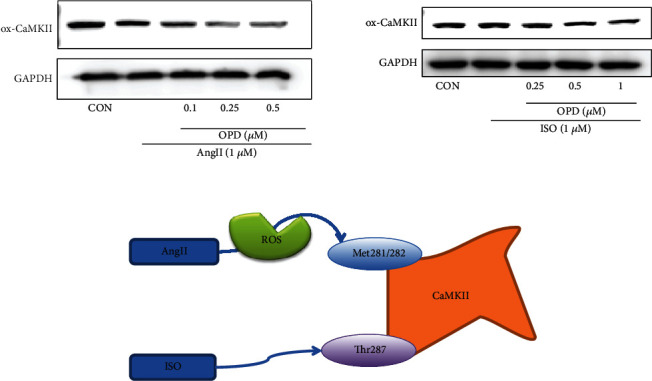
Effect of OPD on CaMKII. OPD activated the phosphorylation of CaMKII in this ISO-induced HF cell model. By contrast, ox-CaMKII was altered very little. Oxidative CaMKII was activated by AngII, whereas OPD released the oxidation.

**Figure 13:**
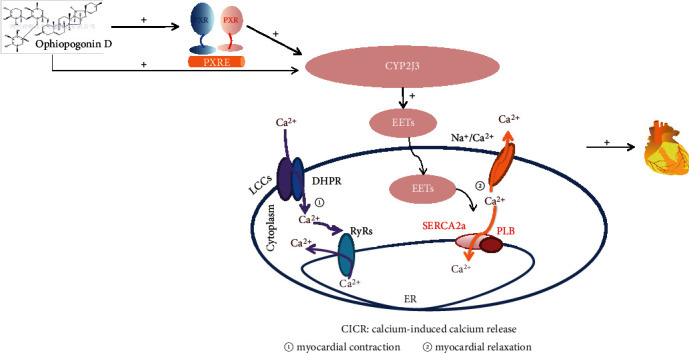
Ophiopogonin D increases SERCA2a interaction with phospholamban by promoting CYP2J3 upregulation (abbreviation: LCCs: L-type calcium channel; EETs: epoxyeicosatrienoic acids; DHPR: dihydropyridine receptor; RyRs: ryanodine receptor; SERCA2a: Ca^2 + ^-ATPase2a; PLB: phospholamban; ER: endoplasmic) reticulum.

In addition, it can be concluded that OPD targets Ca^2+^ homeostasis in the ER. The results of this study revealed that the reduction PLB can restore the frequency response in failing cardiomyocytes [31, 32]. The intracellular Ca^2+^ handling system of the SR plays a critical role in the maintenance of the normal cardiac pumping activity [31, 32]. PLB is recognized as an important regulator for the normal cardiac function [34]. In this study, we demonstrate that OPD associates with downregulation of PLB and upregulation of p-PLB and SERCA2a in two HF cell models (Figures 8(a) and 8(b)). The expression of PLB and SERCA2a is likely related to the size of Ca^2+^ stores in the SR and ultimately influences intracellular Ca^2+^ release and cardiac contractility. Upregulation of PLB may be harmful to cardiac performance by elevating diastolic Ca^2+^ attributable to suppression of SERCA2a. Central regulators of cardiac excitation-contraction coupling are PKA and CaMKII. An interesting phenomenon showed that OPD promoted the phosphorylation of PLB at Ser16 not Thr17 by increasing the PKA activity and no effects on CaMKII in the AngII-induced HF cell model, while in the ISO-induced HF cell model, OPD promoted the phosphorylation of PLB at Thr17 not Ser16 by increasing the CaMKII activity and no effects on PKA. Meanwhile, a series of inhibitors were used to confirm this phenomenon (Figure 9). The study reveals that CaMKII can be activated by oxidation at Met281/282 and phosphorylation at Thr287 [34]. Our results showed that OPD activated the phosphorylation of CaMKII in an ISO-induced HF cell model. CaMKII was phosphorylated by ISO, while OPD influenced the phosphorylation status. By contrast, ox-CaMKII was altered very little. Oxidative CaMKII was activated by AngII, while OPD released the oxidation (Figure 12). Interestingly, there was no obvious change in phosphorylated CaMKII (Figure 9). Previous research has revealed that OPD can clean the ROS to protect endothelial cells from H_2_O_2_-induced oxidative stress [29]. OPD may ease the AngII-mediated oxidative burden to reduce the ox-CaMKII expression. In the heart, autophosphorylation of CaMKII is particularly prevalent during *β*-adrenergic signaling [32, 33]. HF and many of the conditions are associated with significant oxidative stress. ROS have been reported to lead to a dynamic PTM at methionine residues in vitro, which reveals that AngII and endothelin-1 (ET-1) activate CaMKII by a primarily oxidation-dependent pathway [34]. The underlying mechanism may be related to the ISO-induced *β*-adrenergic signaling and AngII-induced primarily oxidation-dependent pathway [35]. We had checked the oxidized and phosphorylated expression of CaMKII in two HF cell models (Figure 11). It is the first time to discover and confirm that OPD has selectivity for PLB phosphorylation sites in heart failure models induced by different factors. Above results indicated that OPD can protect cardiomyocytes by directly promoting the phosphorylation of PKA or CaMKII or by reducing the oxidation level of CaMKII. All data showed that OPD-induced SERCA2a interaction and colocalization with PLB are mediated by CYP2J3 (Figures 3–5). Furthermore, our results support that OPD promotes PXR translocation from cytoplasm to the nucleus (Figures 3–5). PXR siRNA significantly attenuated the expression of CYP2J3 (Figures 10(b) and 10(c)). The PXR was originally discovered as a nuclear receptor for transcription regulation [36]. In our previous study, knocking down PXR might diminished the induced effect of OPD on CYP2J3 [16]. However, since this phenomenon was only initially discovered at that time, the data was not very sufficient at that time. Therefore, in the current study, we continued to verify and further confirmed that PXR was involved in OPD's transcriptional regulation of CYP2J with detailed results. The results further indicated that PXR might participate in the regulation of CYP2J3. Nuclear receptors, including PXR, CAR, and AHR, have regulatory effects on CYP enzyme. It is the first time that has been found and confirmed that PXR participates in the transcription induction of CYP2J. Whether other nuclear receptors or transcription factors participate in the transcription regulation of CYP2J needs further studies. Taken together, our finding that CYP2J3 mediates the interaction between SERCA2a and PLB suggesting that the appropriate expression and function of CYP2J3 is critical in maintaining Ca^2+^ homeostasis. Indeed, alterations in the CYP2J3 expression and translational modification are implicated in the onset and progression of cardiac hypertropy, myocardial I/R injury, and other disordered Ca^2+^ regulation diseases [37–40]. Our laboratory has carried out systematic research around OPD, which has proved that OPD has a very good pharmacological effect on heart failure, mainly by inducing CYP2J to promote the SERCA2a activity, maintaining intracellular calcium homeostasis, and then protecting myocardial cells. It showed that OPD has a good prospect and development value to become a drug for the treatment of cardiovascular disease. Whether it can become a drug for the treatment of heart failure needs to be carried out according to the guiding principle of new drug development, but OPD as the leading compound for the treatment of heart failure is beyond doubt. To this end, our results identify CYP2J3 as an attractive target and OPD as a promising lead for HF therapy (Figure 13).

## Figures and Tables

**Table 1:** Primer sequences used for qPCR.

Gene	Forward primer (5′-3′)	Reverse primer (5′-3′)
CYP2J3	CATTGAGCTCACAAGTGGCTTT	CAATTCCTAGGCTGTGATGTCG
r-PXR	AAAGCAGTGGCCACCTAACA	CCCCACATACACGGCAGATT
GAPDH	CAAGGTCATCCATGACAACTTTG	GGGCCATCCACAGTCTTCTG
PKA	GCTGGCTTTGATTTACGG	GATGTTTCGCTTGAGGATA
CaMKII	CCTGAACCCTCACATCCACC	CCAGGTACTGAGTGATGCGG
SERCA2a	TCTGACTTTCGTTGGCTGTG	GCCTTTGTTATCCCCAGTGA
PLB	TACCTTACTCGCTCGGCTATC	GAGAAGCATCACAATGATGACC

## Data Availability

The [cell viability, measurement of ER Ca^2+^ and Ca^2 + ^-ATPase activity, qPCR, western blot analysis, coimmunoprecipitation assay, immunofluorescence colocalization, small interfering RNA transfection, time-resolved FRET imaging] data used to support the findings of this study are included within the article.

## Abbreviations

FRET: Fluorescence resonance energy transfer
PXR: Pregnane X receptor
OPD: Ophiopogonin D
SERCA2a: Ca^2 + ^-ATPase2a
PLB: Phospholamban.
